# Dissemination of Novel Antimicrobial Resistance Mechanisms through the Insertion Sequence Mediated Spread of Metabolic Genes

**DOI:** 10.3389/fmicb.2016.01008

**Published:** 2016-06-28

**Authors:** Leonardo Furi, Richard Haigh, Zaaima J. H. Al Jabri, Ian Morrissey, Hong-Yu Ou, Ricardo León-Sampedro, Jose L. Martinez, Teresa M. Coque, Marco R. Oggioni

**Affiliations:** ^1^Department of Genetics, University of LeicesterLeicester, UK; ^2^Dipartimento di Biotecnologie Mediche, Universita di SienaSiena, Italy; ^3^IHMA Europe SàrlEpalinges, Switzerland; ^4^State Key Laboratory for Microbial Metabolism and School of Life Sciences and Biotechnology, Shanghai Jiaotong UniversityShanghai, China; ^5^Departamento de Microbiología, Instituto Ramón y Cajal de Investigación Sanitaria, Hospital Universitario Ramón y CajalMadrid, Spain; ^6^Centro de Investigación Biomédica en Red de Epidemiología y Salud Pública (CIBERESP)Spain; ^7^Departamento de Biotecnología Microbiana, Centro Nacional de Biotecnología, Consejo Superior de Investigaciones CientíficasMadrid, Spain; ^8^Unidad de Resistencia a Antibióticos y Virulencia Bacteriana (RYC-Consejo Superior de Investigaciones Científicas)Madrid, Spain

**Keywords:** insertion sequence, transposon, antimicrobial drug resistance, metabolism, resistance risk, fabI, IS5 family, IS1182 family

## Abstract

The widely used biocide triclosan selectively targets FabI, the NADH-dependent trans-2-enoyl-acyl carrier protein (ACP) reductase, which is also an important target for the development of narrow spectrum antibiotics. The analysis of triclosan resistant *Staphylococcus aureus* isolates had previously shown that in about half of the strains, the mechanism of triclosan resistance consists on the heterologous duplication of the triclosan target gene due to the acquisition of an additional *fabI* allele derived from *Staphylococcus haemolyticus* (*sh-fabI*). In the current work, the genomic sequencing of 10 of these strains allowed the characterization of two novel composite transposons TnSha1 and TnSha2 involved in the spread of *sh-fabI*. TnSha1 harbors one copy of IS1272, whereas TnSha2 is a 11.7 kb plasmid carrying TnSha1 present either as plasmid or in an integrated form generally flanked by two IS1272 elements. The target and mechanism of integration for IS1272 and TnSha1 are novel and include targeting of DNA secondary structures, generation of blunt-end deletions of the stem-loop and absence of target duplication. Database analyses showed widespread occurrence of these two elements in chromosomes and plasmids, with TnSha1 mainly in *S. aureus* and with TnSha2 mainly in *S. haemolyticus* and *S. epidermidis*. The acquisition of resistance by means of an insertion sequence-based mobilization and consequent duplication of drug-target metabolic genes, as observed here for *sh-fabI*, is highly reminiscent of the situation with the *ileS2* gene conferring mupirocin resistance, and the *dfrA* and *dfrG* genes conferring trimethoprim resistance both of which are mobilized by IS257. These three examples, which show similar mechanisms and levels of spread of metabolic genes linked to IS elements, highlight the importance of this genetic strategy for recruitment and rapid distribution of novel resistance mechanisms in staphylococci.

## Introduction

The NADH-dependent *trans*-2-enoyl-acyl carrier protein (ACP) reductase FabI is one of the highly conserved enzymes of the bacterial fatty-acids biosynthesis. The FabI enzyme has been recognized as a novel and promising candidate drug target (Payne et al., 2001; Lu and Tonge, 2008) given the absence of a eukaryotic orthologue and its essential role in the growth of bacterial cells (Heath et al., 2001; Ji et al., 2004); a concept recently challenged by the observation that some bacteria do not require biosynthesis of fatty acids during infection of the host (Brinster et al., 2009). Due to the interest in using FabI as a drug target concerns have been raised about the large scale use of the biocide triclosan which targets the active site of FabI (Schweizer, 2001; Hijazi et al., 2016); these concerns also encompass the wider risk that biocide use *per se* may have on antimicrobial drug resistance (Oggioni et al., 2012, 2013, 2015; Coelho et al., 2013; Maillard et al., 2013; Morrissey et al., 2014). In the specific case considered here the concern is due to the fact that resistance to triclosan is in most bacterial species mediated by mutation of the promoter region or coding sequence of *fabI* (Heath et al., 1999; Slater-Radosti et al., 2001; Ciusa et al., 2012; Oggioni et al., 2012; Grandgirard et al., 2015). In *Staphylococcus aureus* about half of resistant isolates have a novel type of triclosan resistance mechanism which is based on the presence of an alternative copy of *fabI* derived from *Staphylococcus haemolyticus* (*sh-fabI*) (Ciusa et al., 2012). In previous work, we reported that the *sh-fabI* gene appears to be part of a 3022 bp transposable element most probably mobilized by a single copy of the insertion sequence (IS) 1272 (Ciusa et al., 2012). IS1272 was originally identified in *S. haemolyticus* during investigation of homology matches to a truncated IS which is part of the *mec* cassette (Archer et al., 1994, 1996; Tonouchi et al., 1994). IS1272 is part of the IS1182 family of insertion sequences (Siguier et al., 2015) and apart from that truncated version found in the *mec* element is absent from *S. aureus*.

It has long been known that IS elements are able to transpose and/or change the expression of nearby or neighboring genes, that they are significantly involved in plasmid and chromosomal recombination, and that they provide the basic structure of many transposons and mobile resistance elements (Siguier et al., 2014, 2015). Soon after the discovery of ISs, it was observed that IS elements were able to transpose resistance genes between replicons (Hedges and Jacob, 1974; Barth et al., 1976). Most of the attention in the more recent sequence based work on antimicrobial resistance has still focussed on acquired “resistance”-genes which confer resistance either by target modification, inactivation of the antimicrobial compound or efflux; this is in part due to the difficulty of defining housekeeping genes identified in high throughput or metagenomic datasets as true “resistance” genes (Martinez et al., 2015). All of this “resistance”-gene oriented work has therefore overshadowed other mechanisms of resistance including those based on heterodiploidy for a metabolic gene (either resistant or susceptible alleles) which, especially in staphylococci, appears to be a mechanism with low fitness cost (Andersson, 2006; Oggioni et al., 2012). The classic example of such heterodiploidy is the presence of an additional copy of a dihydrofolate reductase (*dhfA*) gene on conjugative elements, which thereby confers resistance to trimethoprim; these include the plasmid located *dhfA* gene which is transposed in *E. coli* by Tn7 (Barth et al., 1976), the Tn4003 transposon of *S. aureus* where the *dfrA* gene is mobilized by IS257 (Needham et al., 1995), and the more recently discovered transposable unit that comprises *dfrG* and IS*256* which is located on the Tn*5801* of different species (León-Sampedro et al., 2016). A more recent example in *S. aureus* involves resistance to mupirocin (pseudomonic acid), a potent inhibitor of the isoleucyl tRNA synthetase; this is conferred by an additional plasmid-encoded *mupA/ileS2* gene, which is again mobilized by IS257 (Gilbart et al., 1993; Woodford et al., 1998). With mupirocin, a disinfectant utilized for skin decontamination of MRSA staphylococci, this increased occurrence of *mupA/ileS* genes in clinical settings where mupirocin was used for decolonization has led to changes in disinfection policies (Hetem and Bonten, 2013). Even if the presence of additional *mupA/ileS* genes were found to confer a fitness defect to *S. aureus*, recent modeling experiments have predicted long-term increases in the prevalence of mupirocin-resistant phenotypes (MupR) given the “universal” use of mupirocin (Deeny et al., 2015).

In order to investigate the nature, sequence conservation, epidemiological distribution, and target site specificity of the 3022 bp transposable element carrying the *sh-fabI* gene we have sequenced the genomes of a series of triclosan resistant *S. aureus* isolates (Ciusa et al., 2012) and compared these data to the vast database of published genomes. The aims of this study were to conduct an in depth characterization of the *sh-fabI* carrying element, and to put this work into context with the other resistance mechanisms in *S. aureus* also based on IS mobilization of metabolic genes, which, in this species, appears to be a highly flexible means for the recruitment and rapid spread of novel resistance traits.

## Materials and methods

### Bacterial strains

Sixty-five *S. aureus* strains with reduced susceptibility to triclosan were previously identified by performing standard MIC and MBC assays upon a collection of 1602 clinical isolates (Ciusa et al., 2012; Furi et al., 2013; Grandgirard et al., 2015; Oggioni et al., 2015). Ten out of the 28 isolates carrying the *sh*-*fabI* gene were selected from this collection for this work (Table 1; Ciusa et al., 2012).

**Table 1:** **Relevant information of the ten sequenced *S. aureus* isolates (Ciusa et al., 2012)**.

**Strain**	**TnSha1 insertion site**	**MLST**	**Country**	**Year**	**MIC (mg/L)**	**MBC (mg/L)**	**MRSA**
QBR-102278-1091	D	12	Japan	2002	4	32	MSSA
QBR-102278-1107	G	1	Australia	2002	4	32	MSSA
QBR-102278-1203	E	1	France	2002	2	16	MSSA
QBR-102278-1619	A	8	Spain	2002	4	32	MSSA
QBR-102278-2092	A2	8	Canada	2003	4	32	MSSA
QBR-102278-2210	F	83	Mexico	2003	1	32	MSSA
QBR-102278-2351	A2, and B	3	Brazil	2003	8	32	MSSA
QBR-102278-2365	C	8	Brazil	2003	2	32	MSSA
QBR-102278-2376	C	8	Argentina	2003	4	32	MSSA
QBR-102278-2605	A2	8	Japan	2003	32	64	MRSA

### Whole genome sequencing and bioinformatic analysis

The entire genome of 10 *S. aureus* clinical isolates (Table 1) with reduced susceptibility to triclosan and positive for *sh-fabI* detection by PCR were sequenced as previously described (Ciusa et al., 2012; Table 1). Short reads were assembled using ABySS (British Columbia Cancer Agency, Vancouver, Canada; ver. 1.3.5), improved using the multi-reference based scaffolder MeDuSa (Bosi et al., 2015) and subsequent TnSha1 sequence identification was performed using NCBI's BLAST. 2.3.0+ (Altschul et al., 1997). The sequence of the prototype TnSha1 element corresponds to position 3908 to 887 of GenBank accession JQ712986 relative to *S. aureus* strain QBR-102278-1619. No complete sequence of TnSha2 is present in existing complete genomes in GenBank. One of the plasmid versions of TnSha2, which shows a contig break within *fabI*, corresponds to GenBank accession JCAZ01000023 of *S. aureus* strain M0227 (adzpz-supercont1.20.C23). Genbank BLAST searches for TnSha1 and TnSha2 elements were last accessed in December 2015. DNA secondary structures have been predicted by means of the RNAstructure Web Server (Reuter and Mathews, 2010). TnSha1 targets A through G have been confirmed as gene terminator loops by direct visualization of RNA-seq alignment data retrieved from the NCBI Sequence Read Archive (SRA; http://www.ncbi.nlm.nih.gov/Traces/sra/). More precisely Illumina HiSeq data which were previously generated by sequencing of whole RNA (rRNA depleted) extracted from *S. aureus* ATCC51811 (SRA Experiment ID: ERP005246; SRA Sample ID: ERS421566) (Fagerlund et al., 2014) were aligned to genomes of *S. aureus* COL, GenBank accession no. CP000046, and *S. aureus* MW2, GenBank accession no. BA000033, using BWA-MEM (Li, 2014). The bootstrap maximum likelihood tree was obtained using MEGA6 with default parameters. Sequence Types (ST) were defined using the Multilocus Sequence Typing (MLST) web-based service of the Center for Genomic Epidemiology (Lyngby, Denmark; MLST allele sequence and profile data were obtained from: http://pubmlst.org/; Larsen et al., 2012). Contigs containing the TnSha2 element were screened to identify plasmid sequences by means of the PlasmidFinder web-based service of the Center for Genomic Epidemiology (Ver. 1.3; Lyngby, Denmark).

### Molecular analysis of potential transfer intermediates

Genomic DNA was extracted from *S. aureus* strains in the exponential phase of growth using the High Pure PCR template preparation kit (Roche Diagnostics, Germany). Prior to DNA purification the bacterial strains were grown in MHB (Muller Hinton Broth; Beckton Dickinson) in the presence of sub-MIC concentrations of triclosan (2 mg/L for strain QBR-102278-1619 and 4 mg/L for QBR-102278-2351) or following Mitomycin C induction (1 μg/L) (triclosan PHR1338; Sigma-Aldrich) (Ciusa et al., 2012; Oggioni et al., 2012). Negative controls grown in MHB medium only were also used. A FAM labeled TaqMan probe was designed to detect the circular form of the IS1272 and the composite transposon TnSha1 when both were excised from the chromosome (Table 2). Detection of strains carrying TnSha1 in either the A or B integration sites via real-time PCR amplification was performed in a LightCycler 480 system (Roche Diagnostics, Germany) using primers annealing between the integration site and the transposon (Table 2; Oggioni et al., 2002; Isola et al., 2005; Yesilkaya et al., 2006). Primers LF_30 and LF_31 were used to detect the presence of bacteria with a transposon free integration site A (Table 2).

**Table 2:** **List of primers**.

**Name**	**Position (GenBank ID)**	**Sequence**
LF_26	Taqman probe for TnSha1 (JQ712986)	6FAM-TTCACTTATCCAAGAACTTTATGTCCCGGA-BHQ-1
LF_27	IRL' of TnSha1 (JQ712986)	ATCCTTGCCGGGGTAATACAAC
LF_28	IRL of TnSha1 (JQ712986)	AAAGCGAGCCAACAATACGGAGTA
LF_29	IRR of TnSha1 (JQ712986)	TAGTAGCTCAACGAGCTGAAAATAATC
LF_30	Upstream region flanking the TnSha1 integration site A/A2 (NC_002951)	TTGATTTATTTCCCAGCCTATTCTTTTCA
LF_31	Downstream region flanking the TnSha1 integration site A/A2 (NC_002951)	AGGATGTCGATTTGATTTATATTTTTTGTACAT
LF_32	Downstream region flanking the TnSha1 integration site B (NC_002951)	ATCATTTCGTTTATATATAGCAGACATGATAGA

## Results

**Figure 1:**
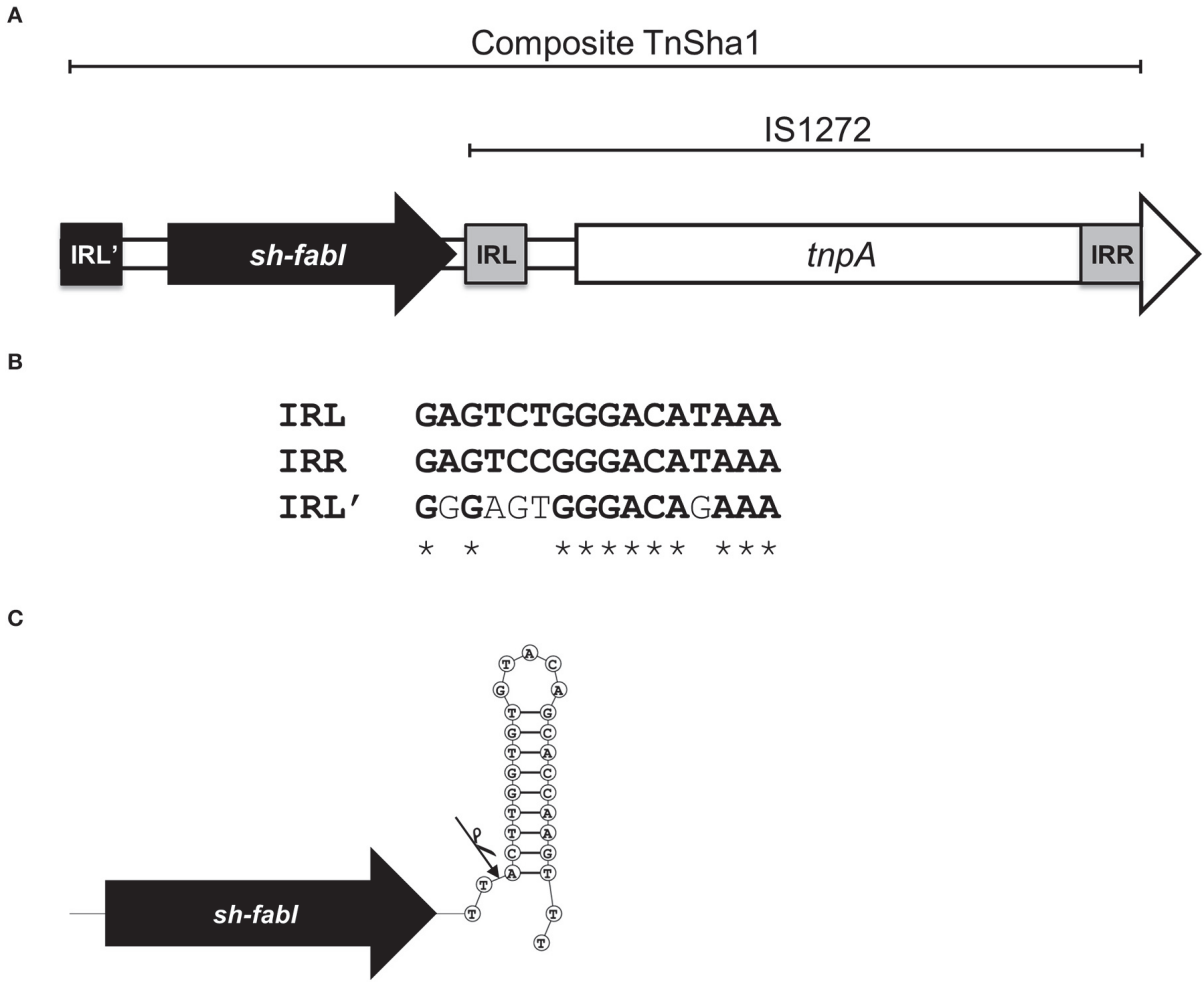
**Structure of the TnSha1 transposon**. The schematic map of the composite transposon TnSha1 in which the *sh-fabI* gene (black) precedes the IS1272 element is shown in panel **(A)**. The updated IS1272 sequences contains six SNPs (four of which indels) with respect to U35635 and encodes for a single transposase gene without a stop codon within the IS element. The sequence of the prototype TnSha1 element corresponds to position 3908 to 887 of Genbank accession JQ712986 relative to strain QBR-102278-1619. Panel **(B)** shows the inverted repeat sequences of IS1272 (named IRL and IRR) aligned to the IRL' upstream of *sh-fabI* which shows 69% identity. Panel **(C)** indicates the hypothesized integration event of IS1272 downstream of *sh-fabI* to generate the prototype TnSha1 element in *S. haemolyticus*. The arrow indicates the insertion point at the base of the *sh-fabI* terminator hairpin.

We have recently reported that a 3022 bp chromosomal element composed of the insertion sequence IS1272 and a *fabI* gene of *S. haemolyticus* (*sh-fabI*) confers resistance to triclosan in *S. aureus* (Figure 1A; GenBank accession no. JQ712986) (Ciusa et al., 2012). The potential for transposition of this unit is conferred by the presence of an alternative inverted repeat (IRL') in the *S. haemolyticus* chromosome upstream of *sh-fabI* with a high degree of similarity to the inverted repeats of IS1272 (Figure 1B). This functional unit, which is composed of the insertion sequence IS1272 and the *sh-fabI* gene, has now being renamed TnSha1 (Figure 1A). It should be noted that none of our genomes, nor any deposited sequence, match the originally deposited sequence of IS1272 (Genbank accession U35635), instead all of the hundreds of IS1272 copies show at least six SNPs (four of which are indels) with respect to U35635 (Archer et al., 1996). These differences mean that the updated IS1272 element encodes for a single transposase gene without a stop codon (Figure 1A).

**Figure 2:**
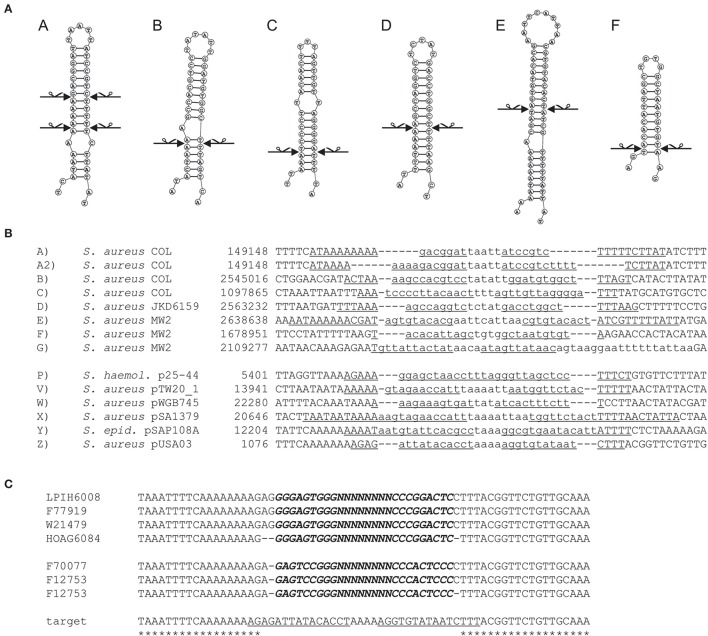
**Insertion sites of TnSha1 in bacterial chromosomes and plasmids**. The seven target sites found in the 10 *S. aureus* strains sequenced in this work have been named from A to G. Staphylococcal plasmid insertion sites are named P to Z. Panel **(A)** shows the hairpin structures formed by the transposon target sites and the scissors indicate the position of sequence breaks generated upon TnSha1 insertion. Secondary structure predictions were made using RNAstructure web-based software (Reuter and Mathews, 2010). Two independent insertions into target **(A)** have been identified and both cleavage sites are shown which in each case generate blunt-end structures in the stem of the hairpin. The sequences in both panels **(A,B)** refer to TnSha1-free target sites in the chromosomes (COL GenBank accession CP000046 and MW2 GenBank accession BA000033) and plasmids. The target sequences in panel **(B)** report in lower case the sequence deleted upon insertion and the inverted repeats underlined. Panel **(C)** shows evidence for multiple insertions of TnSha1 in target Z in the *repA* terminator of plasmids. The aligned sequences show at least four independent insertions in two opposite orientations of TnSha1 into the *repA* terminator in plasmid pUSA03 (target Z). In this panel TnSha1 sequences are shown in bold italics upper case, the deleted part of the target sequence in lower case, and the *repA* terminator hairpin underlined. Note that in panel **(C)** the upper four TnSha1 sequences are in one orientation while the lower three in the opposite orientation. Note that in panel **(C)** the upper four TnSha1 sequences are in one orientation while the lower three in the opposite orientation. The *S. aureus* strain names are given on the right.

**Figure 3:**
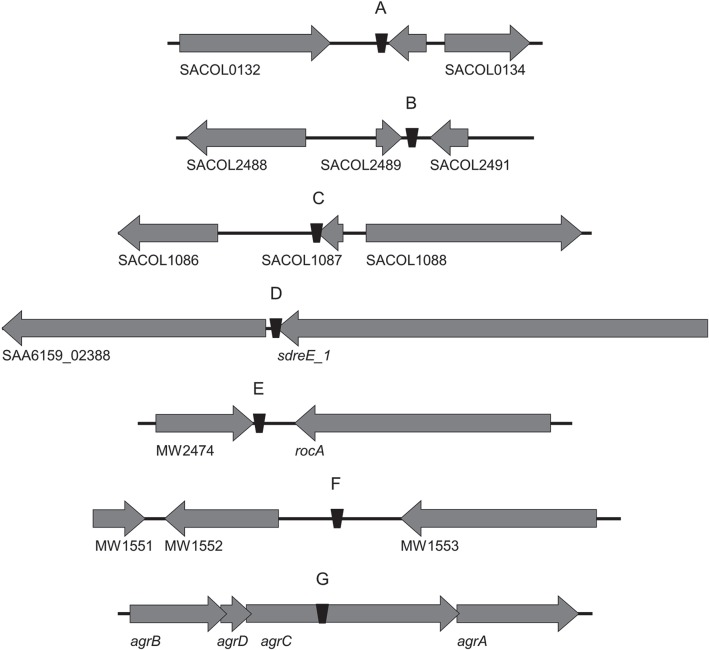
**The seven TnSha1 integration sites found in the sequenced *S. aureus* clinical isolates mapped onto annotated genomes**. The *S. aureus* reference genomes included COL (GenBank accession no. CP000046; **A–C** insertion sites), JKD6159 (GenBank accession no. CP002114; **D** insertion site), and MW2 (GenBank accession no. BA000033; **E,F** insertion sites). 3/7 TnSha1 elements target predicted terminator hairpin structures **(C–E)**, 1/7 targets the *agrC* coding region **(G)**, and 3/7 target intergenic regions **(A,B,F)**. Hairpins **(A)** through **(E)** have been confirmed as transcriptional terminators by mapping RNAseq data.

**Figure 4:**
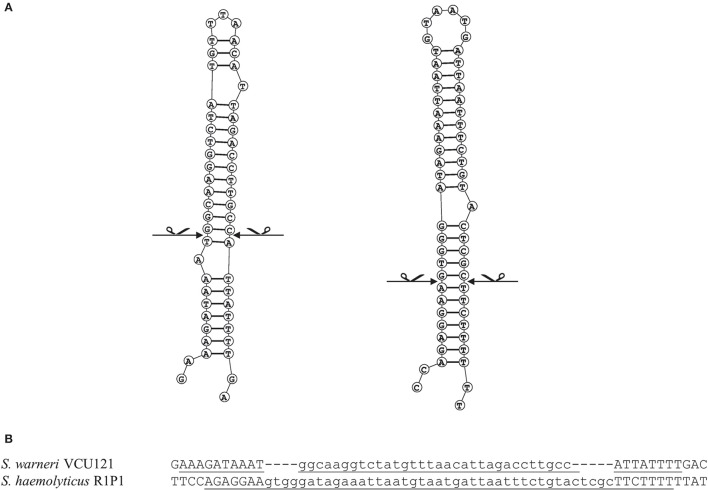
**Analysis of target sites of IS1272 in *S. warneri* and *S. haemolyticus***. Secondary structure predictions of the IS1272 targets in the *S. warneri* strain SG1 (GenBank accession CP003668) are shown in the IS free reference strain VCU121 (GenBank accession NZ_AFEC01000030) (**A** left); and in the *S. haemolyticus* strain Sh29/312/L2 (GenBank accession CP011116) are shown in the IS free reference strain R1P1 (GenBank accession NZ_AJVA00000000) (**A** right). The exact blunt target cleavage sites are indicated by scissors. Panel **(B)** indicates the target sequences showing the inverted repeats (underlined) and deletions (lower case).

In this work the sequence conservation, epidemiological distribution, and target site specificity of TnSha1 were analyzed across a panel of ten sequenced *S. aureus* strains previously reported to carry the *sh-fabI* gene (Ciusa et al., 2012). Sequencing has identified seven different integration sites within the *S. aureus* genomes which have been named A through G (Figure 2, Table 1). The discovery of seven different TnSha1 integration sites in just 10 strains suggests that there is little or no insertion site preference. Analysis of the intact target sites in the reference strains COL or MW2 showed that TnSha1 integration always occurs into hairpin structures and, importantly, that the mode of insertion of TnSha1 always produces a partial deletion of the target sequence (Figure 2B). This mode of integration into the stem of secondary structures is novel to IS elements (Siguier et al., 2015). For one of the targets sequenced in this work there were two different transposition events which could be detected; these lead to deletions of different sizes in the same target site (A and A2; Figure 2B). All seven of the target sites identified by sequencing of our clinical isolates, in addition to other sites subsequently identified in published sequences, were composed of inverted repeat sequences and thus have the potential to form secondary structures with the repeats representing the stems of hairpins (Figure 2A). In each case insertion of TnSha1 produced a partial deletion of the hairpin. This type of cleavage of a secondary structure forming sequences appears to indicate that TnSha1 makes blunt and not staggered cuts, which would have resulted in target duplications. Hairpins A through E have been confirmed as transcriptional terminators by mapping of RNAseq data (Fagerlund et al., 2014; Figure 3). When analysing the target sites of a further 63 complete TnSha1 elements from published *S. aureus* genomes, we identified an additional 12 target sites confirming a lack of any primary sequence specificity within the target. All of the aforementioned features of TnSha1 are consistent with the known behavior of the IS1272 element alone (Archer et al., 1996); i.e., such as the insertion of IS1272 which occurred into the terminator of *sh-fabI* to generate the prototype TnSha1 (Figure 1C), or as in the case of two target sites selected from IS1272 insertions in the genomes of *S. haemolyticus* or *S. warneri* (Figure 4). While most sequenced strains contained just a single copy of TnSha1 in the chromosome, strain QBR-102278-2351 was an exception and carried two TnSha1 elements each in two distinct integration sites (Figure 2, Table 1).

**Figure 5:**
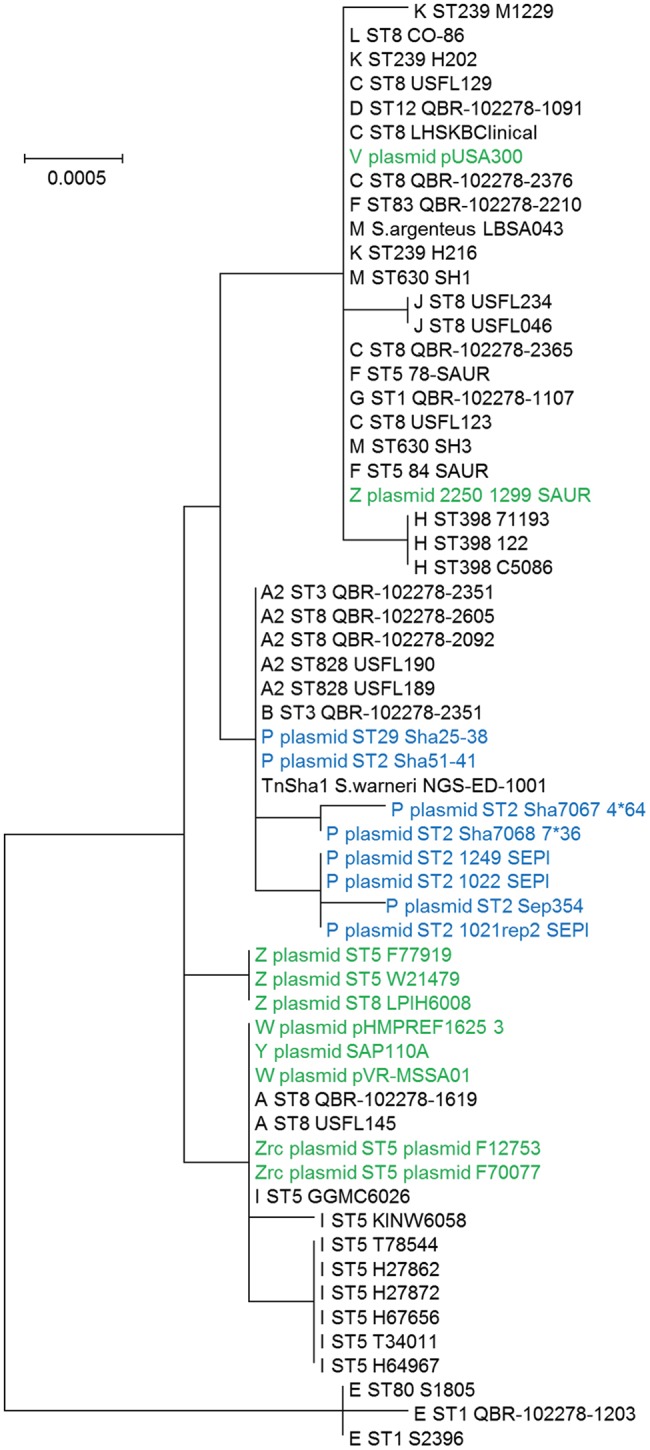
**Phylogram of the TnSha1 and TnSha2 elements**. The tree contains 57 TnSha1 sequences, 10 of which sequenced in this work (QBR-102278 sequences) and a subset of eight TnSha2 sequences (only the homologous part was used for tree building purposes). Each TnSha1 element is identified by its target **(A–Z)**, followed by the sequence type (ST), and the strain name. TnSha1 elements on *S. aureus* plasmids are colored in green while TnSha2 elements are in blue. For GenBank accession numbers please see Tables 1, 4. The bootstrap maximum likelihood tree was obtained using MEGA6 with default parameters. The scale bar is shown in the upper left part. It should be noted that the tree is built based on only few SNPs, which distinguish the different elements.

**Figure 6:**
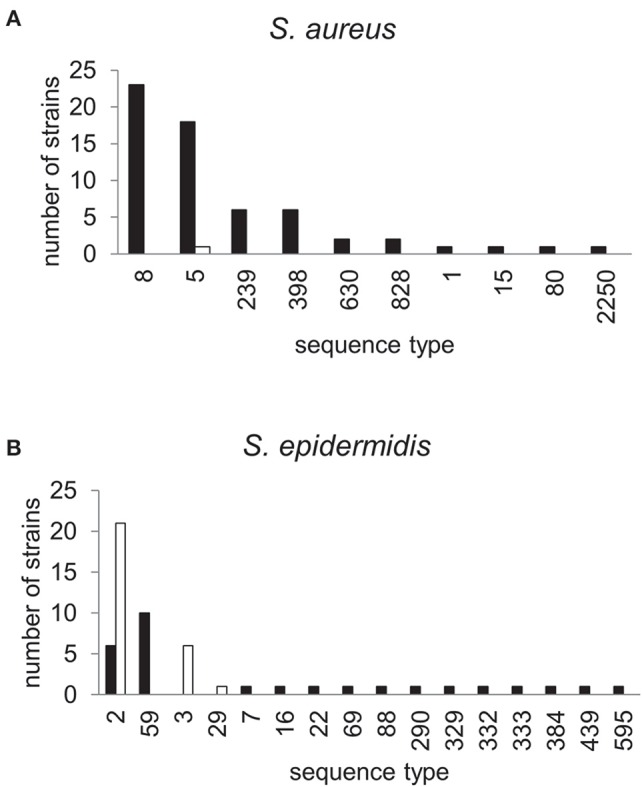
**MLST profiles of staphylococcal strains carrying TnSha1 and TnSha2**. The sequence type (ST) of 62 *S. aureus* **(A)** and 47 *S. epidermidis* strains **(B)** which carry the *sh-fabI* elements TnSha1 (filled bars) and TnSha2 (open bars) are shown. Data are from staphylococcal genomes accessed in GenBank in December 2015 and sequence types were defined using the MLST web-service of the Center for Genomic Epidemiology (Larsen et al., 2012).

The dynamics and epidemiology of transposition between strains was addressed by searching for other TnSha1 elements present in Genbank. Database searches identified 133 complete or partial TnSha1 elements in the genomes of the species *S. aureus, S. argenteus, S. haemolyticus, S. epidermidis, S. saprophyticus*, and *S. warneri*, and in a series of staphylococcal plasmids (Tables 3–5). Of this latter group the integration between *repA* and IS257 in pUSA03 (target Z in Table 4) where TnSha1 was inserted in two different orientations warrants particular attention (Figure 2C). Multi locus sequence typing (MLST) of the strains showed that TnSha1 was present in *S. aureus* in sequence type 8 (*n* = 23), ST5 (*n* = 18), ST239 (*n* = 6), and ST290 (*n* = 6) strains and in *S. epidermidis* in ST59 (*n* = 10) and ST2 (*n* = 6) strains (Table 4, Figure 5). Upon determination of the targets of these newly identified TnSha1 elements it was found that only seven out of the 66 complete TnSha1 elements were integrated into the same seven target sites, A to G, that we had previously identified (Figure 2, Tables 1, 4). Alignment of our previous 10 TnSha1 elements with the seven new TnSha1 elements that were integrated at the same target sites showed clustering of the TnSha1-SNPs (some elements differed by up to 11 SNPs from the consensus) together with their target sites (Figure 6). This pattern is consistent with the insertion of the element into a particular target followed by clonal spread. This scenario appears to be the rule in most cases except for target A2 and Z where the identical TnSha1 element is present in the same target but in strains of different ST (Table 4, Figure 5). To test for TnSha1 mobilization we used PCR with a set of divergent primers on TnSha1, another set on IS1272, and a third set of primers targeting the “A” insertion site. Unfortunately, no excision events producing a circular intermediate could be observed when two *S. aureus* strains were tested with or without the presence of triclosan in the culture medium, and after induction with mitomycin C.

**Table 3:** **BLAST search of staphylococcal genomes for TnSha1 and TnSha2**.

**Species**	**Element**	Chromosomal^*^	Plasmid^**^	**Total**	**Total number of genomes**	**%**
*S. argenteus*	TnSha1	1	1	2	6	33
	TnSha2	–	–	–		–
*S. aureus*	TnSha1	61	3	64	4127	1.5
	TnSha2	1	–	1		0.02
*S. epidermidis*	TnSha1	30	1	31	286	10.8
	TnSha2	19	–	19		6.6
*S. haemolyticus*	TnSha1	3	–	3	141	2.1
	TnSha2	10	–	10		7
*S. warneri*	TnSha1	2	–	2	7	28.6
	TnSha2	–	–	–		–
*S. saprophyticus*	TnSha1	1	–	1	5	20
	TnSha2	–	–	–		–
Total		128	5	133	4567	–

* *The localization designated as chromosomal may also include plasmids present in genome contigs*.

** *Only includes plasmids deposited as complete plasmids*.

**Table 4:** **List of strains carrying the TnSha1 element on the chromosome**.

**Species**	**GenBank ID**	**Strain name**	**Insertion**	**MLST**
			**site**	
*S. argenteus*	CCEM01000001	Sa_LBSA043	M	–
*S. aureus*	AIDT01000009	DR10	H	398
*S. aureus*	AUPV01000018	S100	H	398
*S. aureus*	CAVU010000033	S1805	E	80
*S. aureus*	CAWA010000053	S2396	E	1
*S. aureus*	CFPN01000018	USFL079	L	8
*S. aureus*	CGGX01000004	USFL145	A	8
*S. aureus*	CIAK01000002	USFL234	J	8
*S. aureus*	CIGG01000001	USFL129	C	8
*S. aureus*	CP003045	71193	H	398
*S. aureus*	CSDA01000011	USFL101	L	8
*S. aureus*	CSDP01000002	USFL046	J	8
*S. aureus*	CSEJ01000001	USFL123	C	8
*S. aureus*	CSEV01000003	USFL189	A2	828
*S. aureus*	CSFL01000012	USFL078	L	8
*S. aureus*	CSHX01000003	USFL190	A2	828
*S. aureus*	CSJZ01000023	USFL050	nd	8 slv^*^
*S. aureus*	CTWU01000023	M705	K	239
*S. aureus*	CTWW01000088	H211	K	239
*S. aureus*	CTXO01000001	M1229	K	239
*S. aureus*	CTXZ01000061	H216	K	239
*S. aureus*	CTYD01000081	H202	K	239
*S. aureus*	CVOP01000018	SH3	M	630
*S. aureus*	CVOU01000018	SH1	M	630
*S. aureus*	CVRW01000028	M170	K	239
*S. aureus*	JBFG01000011	KINW6058	I	5
*S. aureus*	JBGB01000006	FVRH6130	nd	8
*S. aureus*	JBGS01000014	GGMC6026	I	5
*S. aureus*	JBLE01000006	SCOA6048	H	8
*S. aureus*	JBMX01000012	SCOA6012	H	8
*S. aureus*	JDLS01000019	T78544	I	5
*S. aureus*	JDOK01000060	F35307	nd	5
*S. aureus*	JDOU01000017	H27862	I	5
*S. aureus*	JDOV01000017	H27872	I	5
*S. aureus*	JDPI01000020	H67656	I	5
*S. aureus*	JECS01000014	T34011	I	5
*S. aureus*	JEDV01000017	H64967	I	5
*S. aureus*	JETJ01000039	T22051	nd	5
*S. aureus*	JGNE01000030	W41757	nd	5
*S. aureus*	JHTT01000046	CO-86	L	8
*S. aureus*	JIXI01000011	PA57	H	398
*S. aureus*	JIYX01000004	C5086	H	398 slv
*S. aureus*	JJDE01000001	122	H	398
*S. aureus*	JURB01000090	84_SAUR	F	5
*S. aureus*	JUTG01000033	78_SAUR	F	5
*S. aureus*	JVUC01000150	1315_SAUR	nd	15
*S. aureus*	JZAL01000002	LHSKBClinical	C	8
*S. aureus*	LAMP01000014	99-06	L	8
*S. aureus*	LAMS01000038	99-48	L	8
*S. epidermidis*	ACHE01000003	BCM-HMP0060	–	59
*S. epidermidis*	AGUC01000094	14.1.R1.SE	–	–
*S. epidermidis*	AHLC01000060	VCU120	–	22
*S. epidermidis*	AHLF01000017	VCU125	–	384
*S. epidermidis*	AKGM01000041	NIHLM067	–	333
*S. epidermidis*	AKGN01000056	NIHLM061	–	332
*S. epidermidis*	AKGW01000049	NIHLM020	–	7
*S. epidermidis*	APHT01000038	528m	–	2
*S. epidermidis*	APHU01000037	41tr	–	2
*S. epidermidis*	ARWU01000031	UC7032	–	595
*S. epidermidis*	JUMV01000141	938_SEPI	–	59
*S. epidermidis*	JUNI01000550	926_SEPI	–	59
*S. epidermidis*	JUVK01000069	73_SEPI	–	16
*S. epidermidis*	JUYJ01000034	655_SEPI	–	59
*S. epidermidis*	JUYK01000190	654_SEPI	–	59
*S. epidermidis*	JVQK01000079	196_SEPI	–	59
*S. epidermidis*	JVSC01000077	154_SEPI	–	59
*S. epidermidis*	JVSC01000077	154_SEPI	–	59
*S. epidermidis*	JVSZ01000046	134_SEPI	–	59
*S. epidermidis*	JVTV01000122	1321_SEPI	–	59
*S. epidermidis*	JWBR01000039	114_SEPI	–	–
*S. epidermidis*	JWCR01000091	1115_SEPI	–	88
*S. epidermidis*	JWEH01000024	1063_SEPI	–	2
*S. epidermidis*	JWFU01000070	1024_SEPI	–	2
*S. epidermidis*	JWFV01000104	1023_SEPI	–	2
*S. epidermidis*	JZUK01000044	NGS-ED-1107	–	2
*S. epidermidis*	JZUL01000024	NGS-ED-1109	–	439
*S. haemolyticus*	CP011116	Sh29/312/L2	–	–
*S. haemolyticus*	CUEZ01000014	CN1197	–	–
*S. saprophyticus*	JXBG01000015	SU8	–	–
*S. warneri*	CANQ01000015	A487	–	–
*S. warneri*	JPOW01000002	NGS-ED-1001	–	–

* *slv = single locus variant; targets A–G from our genome sequences; targets H–Z from Genbank, nd not determined*.

**Table 5:** **List of strains carrying the TnSha1 element on a plasmid**.

**Species**	**GenBank ID**	**Strain name**	**Plasmid**	**Insertion site**	**MLST**
*S. argenteus*	FR821778	MSHR1132	pST75	X2	–
*S. aureus*	AHVE01000013	CIG1165	Plasmid	X	5
*S. aureus*	CP012121	USA300_2014.C02	pUSA300_2014.C02	X	8
*S. aureus*	CP012594	HOU1444-VR	pVR-MSSA_01	W	5
*S. aureus*	CSBT01000019	USFL338	Plasmid	V	8
*S. aureus*	JBGG01000015	LAMC6115	Plasmid	Zrc	8
*S. aureus*	JBJX01000010	SJUD6114	Plasmid	X	8
*S. aureus*	JBRD01000004	AMMC6015	Plasmid	Z	8
*S. aureus*	JBSH01000015	HOAG6084	Plasmid	Z	8
*S. aureus*	JCAZ01000023	M0227	TnSha2	P	5
*S. aureus*	JDXZ01000007	LPIH6008	Plasmid	Z	8
*S. aureus*	JEEN01000028	F77919	Plasmid	Z	5
*S. aureus*	JEOI01000012	W21479	Plasmid	Z	5
*S. aureus*	JEQM01000030	F70077	Plasmid	Zrc	5
*S. aureus*	JGFR01000050	F12753	Plasmid	Zrc	5
*S. aureus*	JGIT01000028	T28653	Plasmid	X	8
*S. aureus*	JICL01000103	880	pHMPREF1625_3	W	–
*S. aureus*	JVUX01000076	1299_SAUR	Plasmid	Z	2250
*S. epidermidis*	GQ900465	SK6536	pSAP110A	Y	–
*S. epidermidis*	JUPD01000185	890_SEPI	TnSha2	P	2
*S. epidermidis*	JUUP01000151	749_SEPI	TnSha2	P	2
*S. epidermidis*	JUYI01000150	656_SEPI	TnSha2	P	2
*S. epidermidis*	JUZG01000070	634_SEPI	TnSha2	P	2
*S. epidermidis*	JUZR01000081	623_SEPI	TnSha2	P	2
*S. epidermidis*	JVBT01000060	568_SEPI	TnSha2	P	2
*S. epidermidis*	JVHA01000086	439_SEPI	TnSha2	P	2
*S. epidermidis*	JVHR01000056	422.rep2_SEPI	TnSha2	P	2
*S. epidermidis*	JVHS01000079	422.rep1_SEPI	TnSha2	P	2
*S. epidermidis*	JVKK01000090	354_SEPI	TnSha2	P	2
*S. epidermidis*	JVXB01000116	1249_SEPI	TnSha2	P	2
*S. epidermidis*	JWBN01000071	1143_SEPI	TnSha2	P	2
*S. epidermidis*	JWDK01000004	1088_SEPI	TnSha2	P	2
*S. epidermidis*	JWFS01000043	1026_SEPI	TnSha2	P	2
*S. epidermidis*	JWFW01000102	1022_SEPI	TnSha2	P	2
*S. epidermidis*	JWFX01000149	1021.rep2_SEPI	TnSha2	P	2
*S. epidermidis*	JWFY01000144	1021.rep1_SEPI	TnSha2	P	2
*S. epidermidis*	JWGH01000004	1013_SEPI	TnSha2	P	2
*S. epidermidis*	JWGL01000037	101_SEPI	TnSha2	P	2
*S. haemolyticus*	CUCK01000025	25-38	TnSha2 in chromosome^*^	P	29
*S. haemolyticus*	CUCN01000051	25-60	TnSha2	P	–
*S. haemolyticus*	CUCZ01000051	51-13	TnSha2	P	3
*S. haemolyticus*	CUDB01000007	51-11	TnSha2 in chromosome	P	3
*S. haemolyticus*	CUDQ01000054	51-33	TnSha2	P	3
*S. haemolyticus*	CUDW01000051	51-41	TnSha2	P	2
*S. haemolyticus*	CUDY01000029	51-43	TnSha2 in chromosome	P	2
*S. haemolyticus*	CUFB01000040	51-15	TnSha2	P	3
*S. haemolyticus*	CUGD01000039	127925	TnSha2 in chromosome	P	3
*S. haemolyticus*	CUGG01000054	113101	TnSha2	P	3

* *Chromosomally integrated TnSha2 elements are reported for clarity in this plasmid table*.

**Figure 7:**
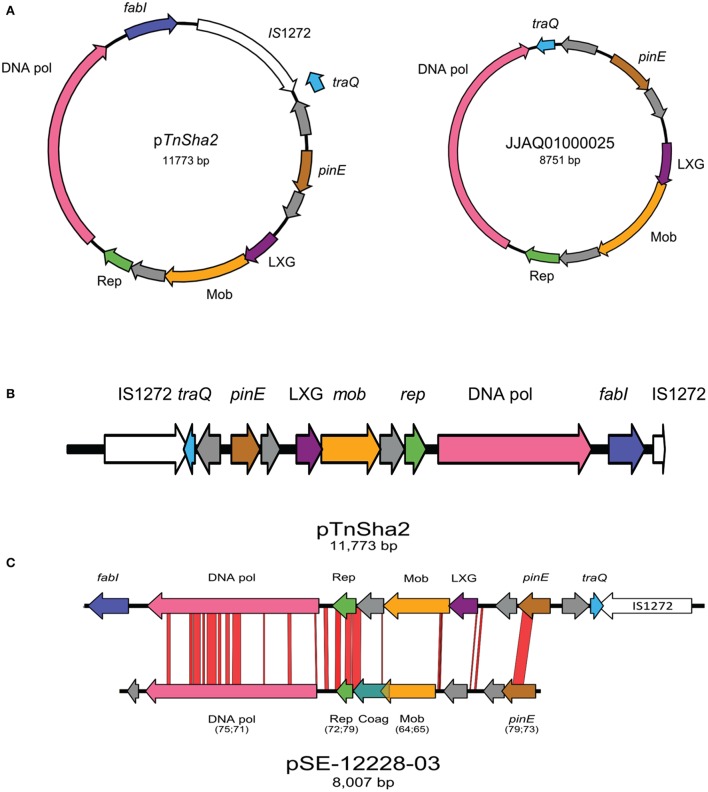
**Schematic map of TnSha2 elements**. The circular maps of the prototype 11.7 kb plasmid sequence of TnSha2 (*S. aureus* strain M0227, GenBank accession JCAZ01000023) and the target 8.7 kb TnSha1-free plasmid (*S. haemolyticus* CUCL01000044 or *S. aureus* JJAQ010000025) are shown in panel **(A)**. The map of TnSha2 integrated into the *S. haemolyticus* chromosome at a position occupied by a single IS1272 in the reference strain is shown in panel **(B)**. In three occasions the resulting chromosomal island is bordered by two IS1272 elements (CUCK01000025 at position 590129 of strain JCSC1435, CUDY01000029 at 1828271, and CUGD01000039 at 2443315) wile in one case only by the “left” IS1272 element (CUDB01000007 at 1322312). Comparison between pTnSha2 element (*S. aureus* JCAZ01000023) and pSE-12228-03 (*S. epidermidis* NC_005006) is shown in panel **(C)**. Numbers between brackets indicate coverage percentage and identity percentage respectively. The genes encode for: a putative DNA-directed DNA polymerase (DNA pol), a replication protein involved in a theta-type replication mechanism (Rep), a putative mobilization protein (Mob), a domain of a group of polymorphic toxin proteins (LXG), a Site-specific DNA recombinase related to the DNA invertase Pin (PinE), and a conjugal transfer pilin chaperone (TraQ).

Within the multiple entries retrieved from Genbank a subgroup showed identical sequences upstream of TnSha1. Investigation of this genomic region showed that in 30 of the strains the TnSha1 element forms part of a larger element (named TnSha2), which in almost all cases is a plasmid, but which can also be found integrated into the genome where it is generally flanked by a second copy of IS1272 (Tables 4, 5). The prototype sequence of TnSha2 (*S. aureus* JCAZ01000023) is an 11.7 kb plasmid in which TnSha1 is inserted into the terminator of *traQ* gene of an 8.7 kb replicon (without fabI and IS1272) present in *S. haemolyticus* (CUCL01000044), but also in *S. aureus* (JJAQ010000025; Figure 7). The *rep* of the plasmid belongs to the Rep_2 family and the DNA polymerase, recombinase and mobilization protein show high sequence identity to those of plasmid p-12228-03 of the *S. epidermidis* reference strain ATCC 12228 (Figure 7; Zhang et al., 2003; Lanza et al., 2015). ThSha2 was detected in the genomes of clonally diverse *S. haemolyticus*, and *S. epidermidis* (ST3 *n* = 6, and ST2 *n* = 21, respectively), but only in a single *S. aureus* (ST5) genome, thereby reflecting different dissemination routes for different species (Table 5). Upon investigating the origin of deposited genome-strains it was observed that the *S. epidermidis* ST2 strains are of hospital origin (Schoenfelder et al., 2010; Roach et al., 2015) and that all of the *S. haemolyticus* strains with TnSha2 and ST3 that we found in this study are clinical isolates (Table 5). Interestingly the database search for IS1272 alone detected no intact elements in *S. aureus*, but did show that about 1/10 of *S. epidermidis* isolates and the majority of *S. haemolyticus* isolates carried IS1272 thereby ensuring that the contigs with the 11.7 kb plasmid in these species are always interrupted in the IS element.

In a phylogenetic tree based on the TnSha1-part of TnSha2, the two elements group into separate branches due to a series of SNPs both in *fabI* and the IS1272 element (Figure 5). In four cases the whole 11.7 kb TnSha2 plasmid appears to be integrated into the chromosome of *S. haemolyticus* in the same position that IS1272 elements are found in reference strain JCSC1435 resulting in a chromosomal island flanked in three cases by two IS1272 copies (CUCK01000025 at position 590.150 of JCSC1435, CUDB01000007 at 1.322.311, CUDY01000029 at 1.828.279, and CUGD01000039 at 2.443.339; Figure 7; Cavanagh et al., 2014).

In the original publication, which described *sh-fabI* in *S. aureus* QBR-102278-1619 (Ciusa et al., 2012), the TnSha1 element was reported to carry a *sh*-*fabI* gene with an A577T SNP with respect to the sequence of the core genome *fabI* of *S. haemolyticus* (Table 6). Since, this SNP had not previously been associated with triclosan resistance of *sa-fabI* in *S. aureus* the gene was reported to represent a susceptible allele (Ciusa et al., 2012). Whilst analysing the panel of *sh-fabI* genes carried by TnSha1 these were all found to show at least one SNP compared to the core-genome triclosan-susceptible sh-*fabI* of *S. haemolyticus* (Table 6). All of these SNPs, except A577T and T578A (see above), have previously been associated with triclosan resistance in the *S. aureus sa-fabI* (Table 6; Ciusa et al., 2012; Grandgirard et al., 2015). The only exception is *S. aureus* strain T22051 which carried a wild type *sh-fabI* gene in TnSha1 thus indicating that the second (or third) IS-element mobilized genomic *fabI* copy generally appears to be a resistant allele.

**Table 6:** ***Sh*-*fabI* nucleotide sequence of 89 TnSha1 and TnSha2 elements**.

**Species**	**Strain**	Polymorphic sites in sh-fabI^*^						Phenotype^**^	**Comment**
			1	5	5	5	6		
		8	0	7	7	9	1		
		1	6	7	8	3	1		
*S. haemolyticus*	JCSC1435	T	G	A	T	C	T	S	Reference strain^***^
*S. haemolyticus*	0281	.	.	.	.	.	.	S	Triclosan susceptible clinical isolate^***^
*S. aureus*	T22051	.	.	.	.	.	.	S	*wt* sequence
*S. aureus*	KINW6058	C	.	T	.	.	.	R	GTA81GCA V27A; ATT577TTT I193F
*S. epidermidis*	354_SEPI	.	A	.	.	.	C	R	GTT106ATT V36I; TC611TCC F204S
*S. epidermidis*	422.rep1_SEPI	.	A	.	.	.	C	R	GTT106ATT V36I; TC611TCC F204S
*S. epidermidis*	422.rep2_SEPI	.	A	.	.	.	C	R	GTT106ATT V36I; TC611TCC F204S
*S. epidermidis*	493_SEPI	.	A	.	.	.	C	R	GTT106ATT V36I; TC611TCC F204S
*S. epidermidis*	568_SEPI	.	A	.	.	.	C	R	GTT106ATT V36I; TC611TCC F204S
*S. epidermidis*	623_SEPI	.	A	.	.	.	C	R	GTT106ATT V36I; TC611TCC F204S
*S. epidermidis*	634_SEPI	.	A	.	.	.	C	R	GTT106ATT V36I; TC611TCC F204S
*S. epidermidis*	656_SEPI	.	A	.	.	.	C	R	GTT106ATT V36I; TC611TCC F204S
*S. epidermidis*	749_SEPI	.	A	.	.	.	C	R	GTT106ATT V36I; TC611TCC F204S
*S. epidermidis*	890_SEPI	.	A	.	.	.	C	R	GTT106ATT V36I; TC611TCC F204S
*S. epidermidis*	1013_SEPI	.	A	.	.	.	C	R	GTT106ATT V36I; TC611TCC F204S
*S. epidermidis*	1021.rep2_SEPI	.	A	.	.	.	C	R	GTT106ATT V36I; TC611TCC F204S
*S. epidermidis*	1022_SEPI	.	A	.	.	.	C	R	GTT106ATT V36I; TC611TCC F204S
*S. epidermidis*	1088_SEPI	.	A	.	.	.	C	R	GTT106ATT V36I; TC611TCC F204S
*S. epidermidis*	1143_SEPI	.	A	.	.	.	C	R	GTT106ATT V36I; TC611TCC F204S
*S. aureus*	F12753	.	.	T	.	.	.		ATT577TTT I193F
*S. aureus*	F70077	.	.	T	.	.	.		ATT577TTT I193F
*S. aureus*	GGMC6026	.	.	T	.	.	.		ATT577TTT I193F
*S. aureus*	H27862	.	.	T	.	.	.		ATT577TTT I193F
*S. aureus*	H27872	.	.	T	.	.	.		ATT577TTT I193F
*S. aureus*	H64967	.	.	T	.	.	.		ATT577TTT I193F
*S. aureus*	H67656	.	.	T	.	.	.		ATT577TTT I193F
*S. aureus*	pHMPREF1625_3	.	.	T	.	.	.		ATT577TTT I193F
*S. aureus*	pVR_MSSA_01	.	.	T	.	.	.		ATT577TTT I193F
*S. aureus*	QBR-102278-1619	.	.	T	.	.	.		ATT577TTT I193F
*S. aureus*	T34011	.	.	T	.	.	.		ATT577TTT I193F
*S. aureus*	T78544	.	.	T	.	.	.		ATT577TTT I193F
*S. aureus*	USFL050	.	.	T	.	.	.		ATT577TTT I193F
*S. aureus*	USFL145	.	.	T	.	.	.		ATT577TTT I193F
*S. epidermidis*	134_SEPI	.	.	T	.	.	.		ATT577TTT I193F
*S. epidermidis*	154_SEPI	.	.	T	.	.	.		ATT577TTT I193F
*S. epidermidis*	196_SEPI	.	.	T	.	.	.		ATT577TTT I193F
*S. epidermidis*	SAP110A	.	.	T	.	.	.		ATT577TTT I193F
*S. aureus*	F77919	.	.	.	A	.	.		ATT578AAT I193N
*S. aureus*	LPIH6008	.	.	.	A	.	.		ATT578AAT I193N
*S. aureus*	W21479	.	.	.	A	.	.		ATT578AAT I193N
*S. aureus*	QBR-102278-1203	.	.	.	.	G	.	R	GCT593GGT A198G
*S. aureus*	S1805	.	.	.	.	G	.	R	GCT593GGT A198G
*S. aureus*	S2396	.	.	.	.	G	.	R	GCT593GGT A198G
*S. argenteus*	pST75	.	.	.	.	.	G	R	TTC611TGC F204C
*S. argenteus*	Sa_LBSA043	.	.	.	.	.	G	R	TTC611TGC F204C
*S. aureus*	122	.	.	.	.	.	G	R	TTC611TGC F204C
*S. aureus*	1299_SAUR	.	.	.	.	.	G	R	TTC611TGC F204C
*S. aureus*	1315_SAUR	.	.	.	.	.	G	R	TTC611TGC F204C
*S. aureus*	71193	.	.	.	.	.	G	R	TTC611TGC F204C
*S. aureus*	78_SAUR	.	.	.	.	.	G	R	TTC611TGC F204C
*S. aureus*	84_SAUR	.	.	.	.	.	G	R	TTC611TGC F204C
*S. aureus*	C5086	.	.	.	.	.	G	R	TTC611TGC F204C
*S. aureus*	CO-86	.	.	.	.	.	G	R	TTC611TGC F204C
*S. aureus*	H202	.	.	.	.	.	G	R	TTC611TGC F204C
*S. aureus*	H216	.	.	.	.	.	G	R	TTC611TGC F204C
*S. aureus*	LHSKBClinical	.	.	.	.	.	G	R	TTC611TGC F204C
*S. aureus*	M1229	.	.	.	.	.	G	R	TTC611TGC F204C
*S. aureus*	QBR-102278-1091	.	.	.	.	.	G	R	TTC611TGC F204C
*S. aureus*	QBR-102278-1107	.	.	.	.	.	G	R	TTC611TGC F204C
*S. aureus*	QBR-102278-2210	.	.	.	.	.	G	R	TTC611TGC F204C
*S. aureus*	QBR-102278-2365	.	.	.	.	.	G	R	TTC611TGC F204C
*S. aureus*	QBR-102278-2376	.	.	.	.	.	G	R	TTC611TGC F204C
*S. aureus*	SH1	.	.	.	.	.	G	R	TTC611TGC F204C
*S. aureus*	SH3	.	.	.	.	.	G	R	TTC611TGC F204C
*S. aureus*	USA300_2014.C02	.	.	.	.	.	G	R	TTC611TGC F204C
*S. aureus*	USFL046	.	.	.	.	.	G	R	TTC611TGC F204C
*S. aureus*	USFL123	.	.	.	.	.	G	R	TTC611TGC F204C
*S. aureus*	USFL129	.	.	.	.	.	G	R	TTC611TGC F204C
*S. aureus*	USFL234	.	.	.	.	.	G	R	TTC611TGC F204C
*S. epidermidis*	1249_SEPI	.	.	.	.	.	G	R	TTC611TGC F204C
*S. epidermidis*	14.1.R1.SE	.	.	.	.	.	G	R	TTC611TGC F204C
*S. epidermidis*	NIHLM020	.	.	.	.	.	G	R	TTC611TGC F204C
*S. haemolyticus*	Sh29/312/L2	.	.	.	.	.	G	R	TTC611TGC F204C
*S. aureus*	QBR-102278-2092	.	.	.	.	.	C	R	TTC611TCC F204S
*S. aureus*	QBR-102278-2351	.	.	.	.	.	C	R	TTC611TCC F204S
*S. aureus*	QBR-102278-2605	.	.	.	.	.	C	R	TTC611TCC F204S
*S. aureus*	M0277	.	.	.	.	.	C	R	TTC611TCC F204S
*S. aureus*	USFL189	.	.	.	.	.	C	R	TTC611TCC F204S
*S. aureus*	USFL190	.	.	.	.	.	C	R	TTC611TCC F204S
*S. aureus*	W41757	.	.	.	.	.	C	R	TTC611TCC F204S
*S. epidermidis*	1026_SEPI	.	.	.	.	.	C	R	TTC611TCC F204S
*S. haemolyticus*	25-38	.	.	.	.	.	C	R	TTC611TCC F204S
*S. haemolyticus*	25-60	.	.	.	.	.	C	R	TTC611TCC F204S
*S. haemolyticus*	51-11	.	.	.	.	.	C	R	TTC611TCC F204S
*S. haemolyticus*	51-13	.	.	.	.	.	C	R	TTC611TCC F204S
*S. haemolyticus*	51-43	.	.	.	.	.	C	R	TTC611TCC F204S
*S. haemolyticus*	51-41	.	.	.	.	.	C	R	TTC611TCC F204S
*S. haemolyticus*	113101	.	.	.	.	.	C	R	TTC611TCC F204S
*S. haemolyticus*	127925	.	.	.	.	.	C	R	TTC611TCC F204S
*S. warneri*	NGS-ED-1001	.	.	.	.	.	C	R	TTC611TCC F204S

* *Polymorphic sites are indicated with respect to the sh-fabI sequence of S. haemolyticus strain JCSC1435*.

** *Phenotype as defined in references: (Ciusa et al., 2012; Grandgirard et al., 2015)*.

*** *These wt S. haemolyticus strains do not carry the TnSha1 element, therefore the fabI sequence reported is the one of the core genome*.

## Discussion

A novel mobile chromosomal element based upon IS1272 and containing the *sh-fabI* gene of *S. haemolyticus* has been identified in a large-scale screen for biocide resistance across more than 1600 *S. aureus* isolates (Ciusa et al., 2012). This element, now named TnSha1, had been found to be present in one third of triclosan resistant strains of *S. aureus* while the other triclosan resistant strains of that study had mutations either within or upstream of the enoyl-acyl carrier protein reductase *fabI* gene of the core genome (Ciusa et al., 2012; Grandgirard et al., 2015). A more detailed analysis of these *S. aureus* strains, and of other available staphylococcal genomes, has shown that the situation is actually more complex. The main finding of this was that *sh-fabI* appears to be transferred by two types of elements; TnSha1, which has a single IS1272 element, and the composite TnSha1-carrying plasmid TnSha2 which can also integrate into chromosomal IS1272 copies generating in most cases an IS1272 bordered island (Figures 1, 7). Similar congruent formations where reported for other similar elements as for example IS257 mobilizing the trimethoprim resistance determinant *dfrA* (Leelaporn et al., 1996). As the two elements do not show any obvious species specific characteristics it was somewhat unexpected to observe such a strong divergence in distribution for these elements, with *S. aureus* almost exclusively harboring TnSha1, *S. haemolyticus* prevalently harboring TnSha2, and only *S. epidermidis* which was found to commonly carry both elements. Neither the GC content (TnSha1 is 31.4%, TnSha2 is 31.8%, *S. aureus, S. haemolyticus* 33%, and *S. epidermidis* 32%), nor the presence of restriction modification systems nor the origin of the isolates can explain this observation; however we admit that this is as yet based on 4127 *S. aureus* genomes, but upon just 286 *S. epidermidis* and 141 *S. haemolyticus* genomes. Similar dynamics are seen with trimethoprim resistance were the housekeeping *dfrA* gene was acquired from other species and successfully transferred by IS257 across different staphylococcal species (Dale et al., 1995; Leelaporn et al., 1996). Whilst not explaining this difference as such, the observation that the intact IS1272 elements are present in most *S. haemolyticus* isolates, only in a few *S. epidermidis* strains and not at all in *S. aureus* already indicates a species specific “behavior” of the IS element. Future experiments on the transposition of the elements in the three species, will hopefully be more successful than the attempted detection of circular intermediates in *S. aureus* and therefore shed light on the observed differences in the epidemiology of the elements.

The mechanism of TnSha1 and IS1272 target recognition and integration appears to be novel. Both TnSha1 and IS1272 are seen to target hairpin secondary structures with little primary sequence conservation. In addition this insertion into the target appears to produce a blunt-end cut leading to insertion of the element and deletion of part of the target (Figures 1–4). This is in line with the original description of IS1272 which reported that the IS did not produce target duplications (Archer et al., 1996). Interestingly the recent review on IS elements by Siguier and colleagues reports that some members of the IS1182 family, of which IS1272 is part, also target palindromic sequences (Siguier et al., 2015).

Albeit that no primary sequence consensus was detected, the hairpin structures targeted by TnSha1 must have some important features in common as we found three targets with evidence for multiple independent insertions i.e., the targets “A” and “X” or the target “Z” where we also observed multiple insertions in both orientations (Figure 2C). The absence of insertion into rolling circle replicating plasmids, coupled to the observation of insertions in opposite directions appear to indicate the absence of an orientation bias observed for IS elements inserting into the replication fork (Siguier et al., 2014). In the case of TnSha2 the situation appears to be more varied. The few available data show that TnSha2 can integrate like IS1272. In addition the few and incomplete genomic data we have could indicate that TnSha2 could be mobilized by the hypothetical ORFs present leading to duplications and co-integrate formation (Needham et al., 1995). The capacity to utilize different integration mechanisms has been described also for other transposons like Tn7 (Siguier et al., 2014).

The large number of both TnSha1 and TnSha2 element sequences available in the database has allowed us to investigate the genesis of this recently generated and spreading element. This was hampered in part by the fact that both elements included IS1272 and that these ISs, like most other repeat sequences, tend to generate contig breaks during the assembly of bacterial genomes. Given the absolute conservation of the position of IS1272 in all of the TnSha1 and TnSha2 elements, we favor the hypothesis that the initial steps leading to the formation of these mobile elements were very few or possibly even only a single event. The initial events in the assembly of the element must presumably have happened in *S. haemolyticus* since the *sh-fabI* derives from the core genome of this species and both elements are also present in this species. It is again not easy to hypothesize any rationale for the almost exclusive presence of TnSha1 in *S. aureus*, nor for the preferential presence of TnSha2 in *S. haemolyticus*. The lack of primary target sequence specificity and the peculiarity of targeting hairpin secondary structures make the elements very versatile and would allow for transposition to many locations within the chromosome and on plasmids. While intact IS1272 itself is not present in *S. aureus* the apparent selection for TnSha1 has very efficiently allowed spread of the element in this species. In the case of TnSha2 the presence of mobilization genes could indicate that this plasmid may be horizontally transferable. Unfortunately, as in the case of the *dfr* genes (Dale et al., 1995; Leelaporn et al., 1996), none of these data provide a solid molecular basis to explain the difference in distribution of the two elements among staphylococcal species. The explanation may however come from a closer look at the structure of the elements themselves. Indeed the very limited range of the diversity in the elements, and the SNPs present in them, indicates they have a recent origin. Another important aspect of the dynamics of spread of this element is related to the different triclosan-resistance conferring SNPs which have accumulated in *sh-fabI.* The presence of triclosan susceptible and resistant alleles in the studied population suggests the first event was acquisition of a susceptible *sh-fabI* allele followed by the selection of resistant alleles after the transfer event by a *fabI* targeting agent, most probably triclosan (Ciusa et al., 2012; Oggioni et al., 2012, 2013; Morrissey et al., 2014; Grandgirard et al., 2015). This sequence of events in which the acquisition of a resistance element is followed by the selection of more efficient alleles is also the basis of the diversification of other resistance elements as plasmids-encoded beta-lactamases (Bush, 2013).

Type II fatty acid metabolism, and in particular FabI, are among the most highly investigated targets for development of antimicrobial compounds (Banerjee et al., 1994; Park et al., 2007; Escaich et al., 2011; Kaplan et al., 2012). This remains true even though there is now debate on the essentiality of bacterial fatty metabolism during invasive infection because, at least for group B streptococci, it has been reported that it is possible to forage host derived fatty acids (Brinster et al., 2009; Balemans et al., 2010). In this respect it is therefore of great interest to monitor the spread of genetic elements which could produce reduced susceptibility to new antibiotics whose action is based on targeting and inhibition of FabI. In this regard it is worth noting that our experimental screening of a collection of 1602 world-wide clinical *S. aureus* isolates detected *sh-fabI* in 24/1602 of strains (1.5%; Ciusa et al., 2012), which matched perfectly with the *in silico* screen of a database of microbial genomes that detected *sh-fabI* carrying elements in 65/4127 *S. aureus* strains (1.57%). This exact overlap in prevalence indicates that genome databases of such size can now serve as suitable datasets for epidemiological investigation, even though databases generally lack detailed background information on strains. While 1.5% may not appear to be a high background resistance level in a population, it could still be a worrying presence when introducing a new FabI-targeting agent. Even worse is the detection of *sh-fabI* elements in 14% of *S. epidermidis* isolates; this appears to reach levels at which clinical use, including the use of triclosan as disinfectant for decontamination, could be jeopardized. It remains possible however that the lower sample size, of <300 genomes screened, and the absence of clinical information on these strains may limit the relevance of this observation.

In order to correlate our data with two well-known examples of IS-mobilizable metabolic genes, we have checked the relative occurrence of the *dfrA-thyE* genes (Rouch et al., 1989), conferring trimethoprim resistance, and *ileS2* genes (Needham et al., 1994), conferring mupirocin resistance, in the same 4800 staphylococcal genomes. BLAST searches for *dfrA-thyE* yielded 279 hits in *S. aureus* and 134 in *S. epidermidis*, while *ileS2* yielded 207 hits in *S. aureus* and 18 in *S. epidermidis*. This compares well with the detection rate in our work of 65 TnSha1/2 elements in *S. aureus* and 50 in *S. epidermidis*. These numbers indicate that IS mediated transfer of metabolic resistance genes, in our case *sh-fabI* genes and triclosan resistance, is a highly relevant mechanism for the acquisition and spread of antibiotic resistance. These data show that it is not only plasmids which serve as vectors of IS mediated resistance gene transfers, but that the spread of composite transposons can also be a highly efficient mechanism for such a goal. A similar well-described mechanisms also exist in staphylococci for the antibiotic resistance genes for example in Tn4001, Tn4002, and Tn4003, (Lanza et al., 2015).

In conclusion our data show that IS mediated transposition of metabolic genes represents a vast and growing antimicrobial resistance phenomenon. In addition to the well-described Tn*4003* element, which mobilizes *dfrA* by way of three IS257 thereby conferring trimethoprim resistance (Rouch et al., 1989), or the IS257 mediated mobilization of *ileS2* conferring mupirocin resistance (Needham et al., 1995), this arsenal now includes TnSha1 and TnSha2; these elements utilize IS transposition, IS-targeted integration and plasmid mobilization to allow transfer of the *fabI* gene of *S. haemolyticus* to different staphylococci and thereby contribute to triclosan resistance and, potentially, to resistance for other FabI-targeting drugs. These data show that IS mobilization of metabolic genes is a powerful and highly flexible mechanism that can very rapidly provide resistance phenotypes to vast numbers of strains and species. In this era where thousands of genomes are readily available in public databases the analysis of such IS mediated mobilization of core genome metabolic genes may warrant a more detailed and larger scale investigation.

## Author contributions

LF performed the genome sequencing and bioinformatic analysis, wrote the manuscript, and approved the final version. RH participated in bioinformatic analysis, wrote the manuscript, and approved the final version. ZA performed wet lab experiments, participated in bioinformatic analysis, participated in the revision of the manuscript, and approved the final version. HO preformed bioinformatic analysis, discussed results and implication and helped in revision of the manuscript and approved the final version. IM had input in the initial study design, participated in the generation and analysis of data, the revision of the manuscript, and approved the final version. RL preformed bioinformatic analysis, discussed results and implication and helped in revision of the manuscript and approved the final version. JM had input in the initial study design, participated in the analysis of data, participated in the revision of the manuscript, and approved the final version. TC had input in the initial study design, participated in the analysis of data, participated in the revision of the manuscript, and approved the final version. MO designed the study, participated in bioinformatic analysis, wrote the manuscript, and is accountable for all aspects of the work.

## Funding

The work was in part supported by EC project KBBE-227258 (BIOHYPO). ZA is funded by a fellowship of the Sultan Qaboos University, Oman.

### Conflict of interest statement

The authors declare that the research was conducted in the absence of any commercial or financial relationships that could be construed as a potential conflict of interest.
